# Encapsulation of Carbon Nanotubes by Styrene and Butyl Acrylate Particles via Suspension Polymerization for Polymerized Toner Applications

**DOI:** 10.3390/ma16113941

**Published:** 2023-05-24

**Authors:** Eid M. Alosime, Omar A. Adam, Ahmed A. Basfar

**Affiliations:** 1 King Abdulaziz City for Science and Technology, P.O. Box 6086, Riyadh 11442, Saudi Arabia; 2 Leibniz Institute of Polymer Research Dresden e.V., Hohe Straße 6, 01069 Dresden, Germany; 3 Mechanical Engineering Department, College of Engineering, King Saud University, P.O. Box 800, Riyadh 11421, Saudi Arabia; abasfar@ksu.edu.sa; 4 Nuclear Engineering Program, College of Engineering, King Saud University, P.O. Box 800, Riyadh 11421, Saudi Arabia

**Keywords:** suspension polymerization, toner, carbon nanotubes, charge control agent, conversion

## Abstract

Electrophotographic printing and copying processes primarily use toner, which is a mixture of colorant, polymer, and additives. Toner can be made using traditional mechanical milling techniques or more contemporary chemical polymerization techniques. Suspension polymerization provides spherical particles with less stabilizer adsorption, homogeneous monomers, higher purity, and easier control of the reaction temperature. In contrast to these advantages, however, the particle size resulting from suspension polymerization is too large for toner. To overcome this disadvantage, devices such as high-speed stirrers and homogenizers can be used to reduce the size of the droplets. This research investigated the use of carbon nanotubes (CNTs) instead of carbon black as the pigment in toner development. We succeeded in achieving a good dispersion of four different types of CNT, specifically modified with NH_2_ and Boron or unmodified with long or short chains in water rather than chloroform, using sodium n-dodecyl sulfate as a stabilizer. We then performed polymerization of the monomers styrene and butyl acrylate in the presence of the different CNT types and found that the best monomer conversion and largest particles (in the micron range) occurred with CNTs modified with boron. The insertion of a charge control agent into the polymerized particles was achieved. Monomer conversion of over 90% was realized with all concentrations of MEP-51, whereas conversion was under 70% with all concentrations of MEC-88. Furthermore, analysis with dynamic light scattering and scanning electron microscopy (SEM) indicated that all polymerized particles were in the micron size range, suggesting that our newly developed toner particles were less harmful and environmentally friendly products than those typically and commercially available. The SEM micrographs clearly showed good dispersion and attachment of the CNTs on the polymerized particles (no CNT aggregation was found), which has never been published before.

## 1. Introduction

Toner development has attracted the attention of many researchers. Carlson registered the first toner patent in 1938, and in 1960 the first Xerox automatic reproduction machine, which used a dry printing process, was invented. In 1978, this fine-tuned technology was applied to the printing needs of business, and efforts have been made to improve toner development since then [1,2]. These developments have gone through many stages. Earlier, toner was primarily produced by mixing the ingredients in an extruder at a high temperature, and this is known as pulverized toner. More recently, toner particles have been produced through coupling by chemical reaction, and these polymerized particles are smaller and more uniform than those in conventional toner [3]. Compared with the traditional approach, a polymerized toner obtains a sharper print and is more environmentally friendly because less toner is used. 

Suspension [4], emulsion [5,6], dispersion [7], interface/free radical, and aggregation polymerization are the patented methods for toner preparation. Among these techniques, the suspension method has the advantage of preparing toner particles with a perfectly spherical shape and which absorb less stabilizer than in the other approaches [4]. However, suspension polymerization typically produces particles greater than 5 μm, which is significantly larger than ideal. To overcome this disadvantage, devices such as magnetic or mechanical high-speed stirrers and homogenizers are employed to reduce the size of the droplets. However, agitation speed, the nature and quantity of the suspension stabilizer, and the volume ratio of the dispersed (organic) and continuous (aqueous) phases can all affect the size of the monomer droplets. This phase of our work mainly concerns the development of toner technology using polymerization, and so we are particularly focused on suspension polymerization. However, the suspension technique is not new, having been invented more than 80 years ago, with Bauer and Lauth accomplishing the first suspension polymerization—of acrylic monomers that formed beads—in 1931 [8]. 

Santos et al. [9] state that suspension polymerization is a reaction via free radicals in a heterogeneous medium. Furthermore, suspension polymerization occurs in a liquid (often water) phase, where neither the monomer nor the polymer is soluble. The most common type of suspension technique is powder polymerization, in which the polymer is insoluble in its monomer and consequently precipitates out, producing uneven grains or particles. PVC is an excellent example of powder suspension polymerization. Bead suspension is the second form of this kind of polymerization, in which the polymer is soluble in its monomer, creating smooth spherical particles.

To impart a charge to the toner particle, a charge control agent (CCA) is introduced to the toner [10]. CCAs are frequently ion surfactants or metal soaps that produce inverse micelles in the liquid dispersant [11]. Toner materials or particles can be charged positively or negatively depending on the particle materials and CCA employed [12]. Christie [13] defines CCAs as compounds that aid the management of the electrostatic charge applied during the printing process. Ionic materials (either anionic or cationic) can be colored or uncolored. The CCA imparts a positive charge to the toner particles [14,15]. Quaternary ammonium and diazo-type compounds can be used as CCAs [16]. However, some charge control additives are incompatible with thermoplastic toner resins, or they adversely affect the electrical properties of the polishes. In addition, CCAs with low molecular weight may leach out some toner composition and contaminate the carrier’s surface. Improved toner composition uses partially quaternized vinyl pyridine polymer as the CCA [17,18,19].

According to Michel et al. [20], there are charge additives, charge aides, and charge-directing agents in addition to CCAs. Moreover, there is a class of compounds that can stabilize the triboelectric charge in matrix systems, such as electrophotographic toners [21], and these substances are known as charge stabilizers. Charge stabilizers have lower charging magnitudes but more robust long-term charge stability than CCAs. A charge stabilizer can be used within a matrix system or as an external blended addition [22]. Higashiyama et al. [23] report the effect of an externally added CCA on the contact charge between polymers, namely, low-density polyethylene (LDPE) and high-density polyethylene (HDPE), with a diameter of around 5 mm, concluding that the CCA makes LDPE charge positively and HDPE charge negatively. Nie et al. [24] describe the preparation of poly(styrene-co-butyl acrylate)-encapsulated single-walled carbon nanotubes (SWCNTs) under ultrasonic irradiation. First, they chemically modified the SWCNTs by grafting 3-(trimethoxy)-propyl methacrylate-silane (silane-coupling agent, KH750) onto the surface of the SWCNTs. In the second step, they initiated in situ emulsion polymerization of monomer styrene (St) n-butyl acrylate (nBA) in the presence of KH570-g-SWCNTs using ultrasonography. As a result, a poly (St-nBA)/SWCNT emulsion was created. The transmission electron microscopy (TEM) micrographs in that study, however, indicated that the SWCNTs had been coated with the produced polymer.

The objective of the present paper is to use CNTs as a pigment instead of carbon black (CB) for toner applications. To achieve this goal, suspension polymerization was selected as the best way to produce latex of micron size that is well suited to encapsulate CNTs. The main challenges to reaching our objective are the dispersion of CNTs, the insertion of the CCAs in the polymerized latex, and the reduction in the retarding/inhibiting effects of mainly carbonyl groups when CB is used.

## 2. Materials and Methods

### 2.1. Materials

Styrene (St), 99% (Duksan pure chemical, Seoul, Republic of Korea) and n-butylacrylate (nBA), 99% (Samchun pure chemical co., Ltd., Gyeongg-do, Republic of Korea) were used as monomers without further purification. Benzoyl peroxide (BP), 97% (Alfa Aesar, Ward Hill, MA, USA) and divinylbenzene (DVB), 80% (Merck, Hohenbrunn, Germany) were used as the initiator and a crosslinking agent, respectively. Polyvinyl alcohol (PVA; 87–89% hydrolyzed, high-molecular-weight; Alfa Aesar, Ward Hill, MA, USA) was used as the stabilizer. Sodium n-dodecyl sulfate (SDS; International Laboratory, South San Francisco, CA, USA) was used as the dispersant (as suspension stabilizer). Ethanol, 99% (Fisher Chemical, Fair Lawn, NJ, USA) was used as the solvent, and distilled water was used as the dispersion medium.

The carbon nanotubes (CNTs) used in the current work were multi-walled CNTs (MWCNTs) with lengths of 0.5–12 µm and an average diameter of 3.0–30 nm. Four types of CNT were investigated: (1) long and (2) short carboxylated, (3) aminated, and (4) boron-incorporated. The CNTs were used as received (purity >90%; Grafen Chemical Industries, Ankara, Turkey), and their properties are listed in Table 1.

Two types of CCAs were obtained from KMT Co. Ltd., Republic of Korea, and their properties are listed in Table 2. The CCAs were chosen from the group comprising benzyl-tributyl-ammonium 4-hydroxy-naphthalene-1-sulfonate (MEP-51) and aluminum salicylate (MEC-88).

### 2.2. Methods

#### 2.2.1. Polymerization of Poly (St-nBA) in the Presence of CNT-D and CCA (MEC-88)

Purified monomers of styrene and nBA were mixed with DVB (as a cross-linker) and BP (as an initiator) to form a dispersed phase. MEC-88 was then added to the dispersed phase, and the full amount was dissolved. The continuous phase was prepared by dissolving PVA with a molecular weight of 13,000 g/mol in distilled water while heating the mixture at 70 °C under gentle stirring; SDS (as a suspension stabilizer) was added to the solution. The dispersion of CNT-D was performed by sonication under ice (to decrease the temperature generated by the sonicator) for 10 min at an amplitude of 75% in the aforementioned continuous phase.

**Figure 1:**
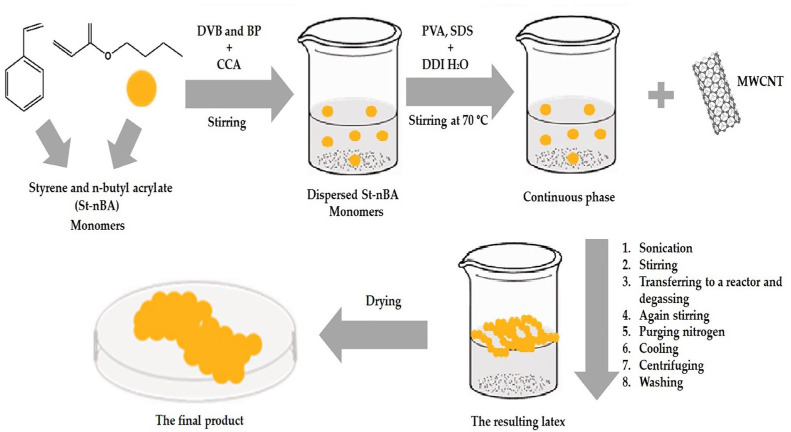
A schematic illustration of suspension polymerization of poly (St-nBA) in the presence of CNTs and CCA.

A continuous phase containing well-dispersed CNT-D was stirred with a T 25 Digital Ultra-Turrax (IKA, Staufen, Germany) for 10 min at a speed of 7000 rpm. While being stirred, the dispersed phase (monomers + BVB + BP) solution was added drop by drop to produce a suspension. The mixture was transferred to the reactor and degassed by vacuum at 70 mbar for 10 min. The mixture was then stirred again by a mechanical stirrer (Eurostar 100 Control; IKA, Staufen, Germany) at a speed of 180 rpm. Finally, the suspension was polymerized at 75 °C for 7 h under the slow purging of nitrogen gas for deoxygenation. The reaction was terminated by cooling at room temperature. The resulting latex was dispersed in distilled water and isolated by centrifugation. The supernatant was decanted, and the remaining polymer was washed again; after repeating the washing step, the supernatant was clear. Finally, the product was dried in a vacuum oven for 24 h without heating to remove any residual water, as shown in Figure 1. The recipes for all the experiments are shown in Table 3.

#### 2.2.2. Polymerization of Poly (St-nBA) in the Presence of CNT-D and CCA (MEP-51)

The purified monomers DVB and BP were prepared as before to produce the dispersed phase. In a deviation from the previous procedure, MEP-51 was not added directly to the dispersed phase because there was no likelihood of it being dissolved in any of the monomers. We instead tried to dissolve it in different amounts of either acetone or ethanol and then mixed it into the continuous phase in the same way as in the procedure for MEC-88. The remaining steps were the same as for MEC-88. The recipes for all the experiments are shown in Table 4 and Table 5.

### 2.3. Characterizations

#### 2.3.1. Monomer Conversion and Solid Content

Monomer conversion was determined gravimetrically. After adding the hydroquinone solution to the latex, the samples were placed in a Petri dish and dried overnight under a hood. The Petri dish was then transferred into a vacuum oven without heat, and the drying process was continued until the weight of the sample was constant. Three measurements were performed for every sample.

$$Solid\;content\;\left(\%\right)=\frac{D_{L}}{L}\times 100$$

$$Monomer\;conversion\;\left(\%\right)=\frac{\left[\frac{D_{L}}{L}\times T_{L}\right]-T_{NV}}{T_{M}}\times 100$$

where *D_L_* = the weight of the dry latex in the Petri dish; *L* = the weight of the latex taken in the Petri dish; *T_L_* = the weight of the total latex; *T_NV_* = the weight of the nonvolatile material other than the monomer; and *T_M_* = the weight of the monomer.

#### 2.3.2. Dynamic Light Scattering (DLS)

Nano-ZS (Malvern Co., Malvern, UK) with a measurement range from 0.3 nm to 10 microns (diameter) was used. A He-Ne laser light source (633 nm, Max 5 mV, minimum sample volume 12 μL) was used to measure the particle size of the prepared latex. Further, the samples were prepared in 0.01 N NaCl solution according to the Malvern recommendation for latex standard.

#### 2.3.3. Scanning Electron Microscopy (SEM)

SEM (NNL 200; FEI Company, Eindhoven, The Netherlands) was used to characterize the morphology of the polymerized latex. For this purpose, a drop of diluted polymerized latex (1:5) with Millipore water was spread to dry over the copper holder, and this drop was dried in air and metalized with 3 nm Pt.

## 3. Results and Discussion

### 3.1. Effect of CNT Types and CNT Concentrations on Suspension Polymerization of Styrene and nBA

First, the effect of the four types of CNTs (nonmodified multiwalled CNT (CNT-A), nonmodified short chain CNT (CNT-B), modified CNT with NH_2_ (CNT-C), and modified CNT with boron (CNT-D)) on the suspension polymerization of styrene and nBA was examined. The findings showed that the maximum conversion of monomers and the largest particle size were both achieved when CNT-D was used, as shown in Table 6.

CNTs modified with amino groups have been shown to have enhanced compatibility with polar polymers because of hydrogen bonding, and CNTs modified with boron can have improved electrical properties through the introduction of a p-type doping effect. The existing literature shows that the interaction of boron and nitrogen atoms with hydrogen atoms and the increased surface area and defective sub-structures within the structure due to the modification plays a vital role in the increase in hydrogen adsorption [25]. The active species for hydrogen adsorption thus appear to be structural defect points formed on the surface due to the increased surface area following modification. The functional groups on the CNT surface are more likely to interact with primary or propagating radicals, leading to retarding/inhibiting effects, which can be used to explain this finding [26]. When peroxy compounds are used as initiators, the main radicals interact with the CNTs, and the CNTs can thus cause the peroxy initiator to decompose, even at low temperatures. Undoubtedly, an oxidation process is taking place in this interaction, and strong oxidizing substances, such as peroxy species, cause the CNT surfaces to oxidize. An increase in functionalities also increases the inhibitory impact when the surface functional groups of the CNTs are taken into account. To determine whether this finding is valid for all concentrations, an assessment was made of the impact of different CNT concentrations on the conversion and polymerized particle size for all four CNT types, the results of which are listed in Table 7.

These results show that using modified CNTs in suspension polymerization can significantly affect how well monomers convert and the size of the polymer particles produced. Specifically, using a modified CNT treated with boron was found to significantly impact the process, producing the highest conversion rate and largest particle size. This is consistent with previous research that showed that modifying CNTs can influence the properties of the polymers produced using different polymerization methods [27]. Li et al. [27], for example, found that functionalized CNTs (CNT-OH or CNT-COOH) were more homogeneously dispersed in a styrene–acrylic matrix and produced stronger interfacial adhesion with styrene–acrylic macromolecules, with the neat styrene–acrylic solid content reaching 42.80% and the monomer conversion rate reaching 96.20%. Park et al. [28], meanwhile, reported that modifying CNTs with carboxyl groups improved their dispersion in a polymer matrix, leading to enhanced mechanical properties in the resulting composite. Comparing these results with functionalized CNT-reinforced composites, they found that a higher aspect ratio was essential for improving strength [27,28]. Kim et al. [29] determined that modifying CNTs with different functional groups can significantly affect their properties and, consequently, their interactions with polymers. From Table 7, it is clear that the conversion of polymerized particles did not rise above 78% for nonmodified CNTs, which indicates that the carbonyl group still has retarding/inhibiting effects on suspension polymerization, which is in line with the existing literature [26]. The carbonyl group’s inhibition/retardation of the suspension polymerization stems from its interaction with free radicals generated during the polymerization process [30]. The carbonyl group acts like a trap, capturing free radicals and preventing them from interacting with additional monomer molecules, which slows down the overall polymerization rate, thus explaining the conversion rate failing to exceed 78%.

In addition, by forming stable cross-linked polymer networks, carbonyl groups can impact suspension polymerization. This process forms an insoluble polymer, reducing the polymer conversion efficiency [31,32]. Furthermore, these insoluble networks trap monomers and prevent them from reacting, leading to slower conversion rates and lower polymer conversion efficiency, as shown in Table 7. Hardy et al. [33] found that increasing the carbonyl-functionalized comonomer in a polymerization mixture led to a decrease in the polymer yield because of the formation of insoluble networks. Similarly, Hedayati et al. [34] found that carbonyl groups in a polyvinyl alcohol stabilizer used in the suspension polymerization of styrene resulted in the formation of an insoluble network, which decreased conversion efficiency, leading to lower polymer yields.

Table 7 also presents CNTs modified with NH_2_ and boron. Retarding/inhibiting effects remained for CNTs modified with NH_2_, even though most of the carbonyl groups were replaced by NH_2_ (it was not possible to replace all of them). Despite the findings presented in Table 7, further research is required to comprehensively understand the underlying mechanisms responsible for the observed effects and to offer a sound explanation. Several contributing factors could be at play, including the particular structure of the modified CNTs, how they interact with the monomers, and the overall kinetics of the polymerization process, and it is essential to consider all these variables. According to Chen et al. [35], CNTs modified with amino groups have greater compatibility with polar polymers because of the presence of hydrogen bonding, while CNTs modified with boron have improved electrical properties because of the introduction of a p-type doping effect. This explains why the best conversions were achieved with CNTs modified with boron, as shown in Table 7; in all cases, the conversions were 90% or above, and it is thus clear that there were no retarding/inhibiting effects. Other interesting results were found with particle sizes in the micron range; of all the concentrations tested, the one with 0.25 wt% proved the best for obtaining encapsulated CNTs by suspension-polymerizing styrene and nBA.

### 3.2. Suspension Polymerization of Styrene and nBA in the Presence of CNT-D and CCA (MEC-88) in Different Concentrations

Kedzior et al. [36] reported that suspension polymerization is a widely used technique for producing latex particles, which have a broad range of applications in coatings, adhesives, and toners. nBA and styrene are two commonly used monomers in suspension polymerization, and their copolymers have been widely used in toner applications because of their high charging characteristics and excellent print quality [37]. Since the present work aims to develop new materials for toner applications, CNTs should be either encapsulated or attached to the polymerized particles. According to Bhanvase and Sonawane [38], CNT encapsulation—being attached to the polymerized particles—is necessary to improve the particles’ dispersion and enhance their electrical conductivity. A CCA must also be inserted in polymerized particles or stuck on the surface [39] to improve the charging characteristics of the resulting toners. In the present study, MEC-88, which is a negatively charged CCA type, was dissolved in styrene before polymerization took place to ensure its even distribution throughout the polymerization process.

**Figure 2:**
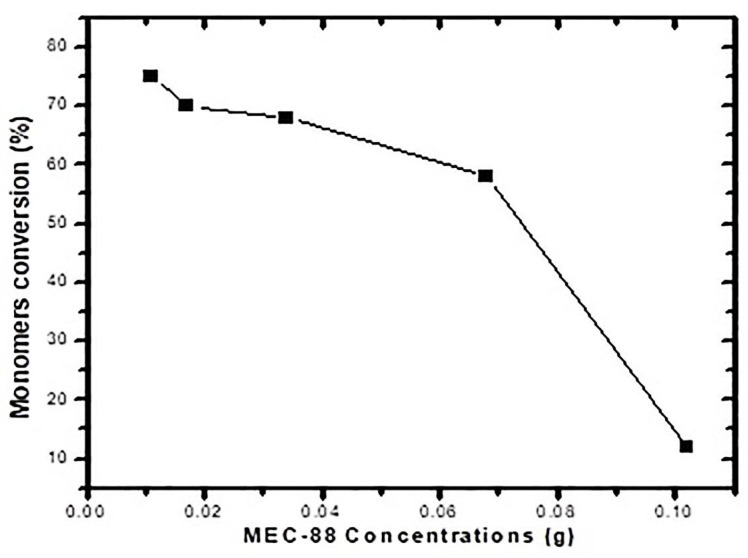
Monomer conversion versus MEC-88 concentration for St and nBA.

Table 8 presents the particle size, measured by DLS, for converting polymerized particles using MEC-88 concentrations ranging from 0.05 wt% to 0.42 wt%, and Figure 2 plots the conversions against the MEC-88 concentrations. It is evident that conversion decreased with greater MEC-88 concentrations, implying an inverse relationship between them. Figure 2 shows that the maximum conversion (75%) was achieved with the smallest concentration of MEC-88 (0.05 wt%), but the conversion surprisingly did not increase to more than 75% in any case, making the conversion undesirable for toner material production.

**Figure 3:**
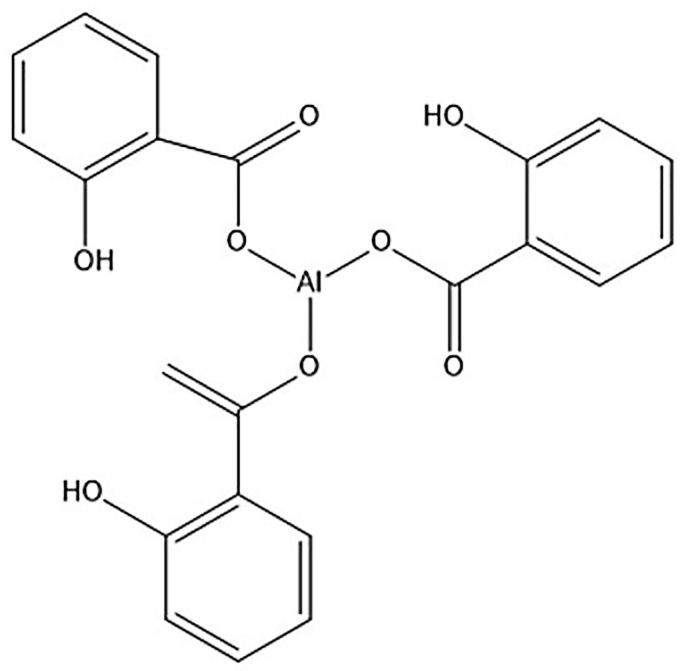
Chemical character of MEC-88.

It is well known that the presence of CB retards or inhibits most types of radical polymerization because it is a potent radical scavenger [40]. In addition, the functional groups on the surfaces of CB (mostly the carbonyl group) interact with primary or propagating radicals, resulting in reaction retardation/inhibition [31,37]. More details on the retarding/inhibiting effects of the carbonyl group were reported by Kiatkamjornwong et al. [26], but to relate this observation to the phenomena discussed in the previous paragraph it would be fruitful to look at the chemical characteristics of MEC-88 (see Figure 3).

From Figure 3, it can be seen that MEC-88 has a carbonyl group. Maiti et al. [41] described how carbonyl groups harbored in a chemical structure likely interact with radicals during the polymerization process, thereby retarding or inhibiting it. This interaction could explain the decrease in the conversion rate observed with increased MEC-88 concentrations in the current study. The findings show that obtaining a high conversion of monomers in the presence of MEC-88, regardless of the CCA improving the charging characteristics of the toner, is not possible; hence, there is a trade-off when increasing MEC-88 concentrations between the conversion rate and achieving desirable electrical conductivity and toner charging characteristics. As this research shows, suspension polymerization of nBA and styrene in the presence of CNT-D and CCA improves the electrical conductivity and charging characteristics of the resulting toners but produces a low conversion rate in return.

**Figure 4:**
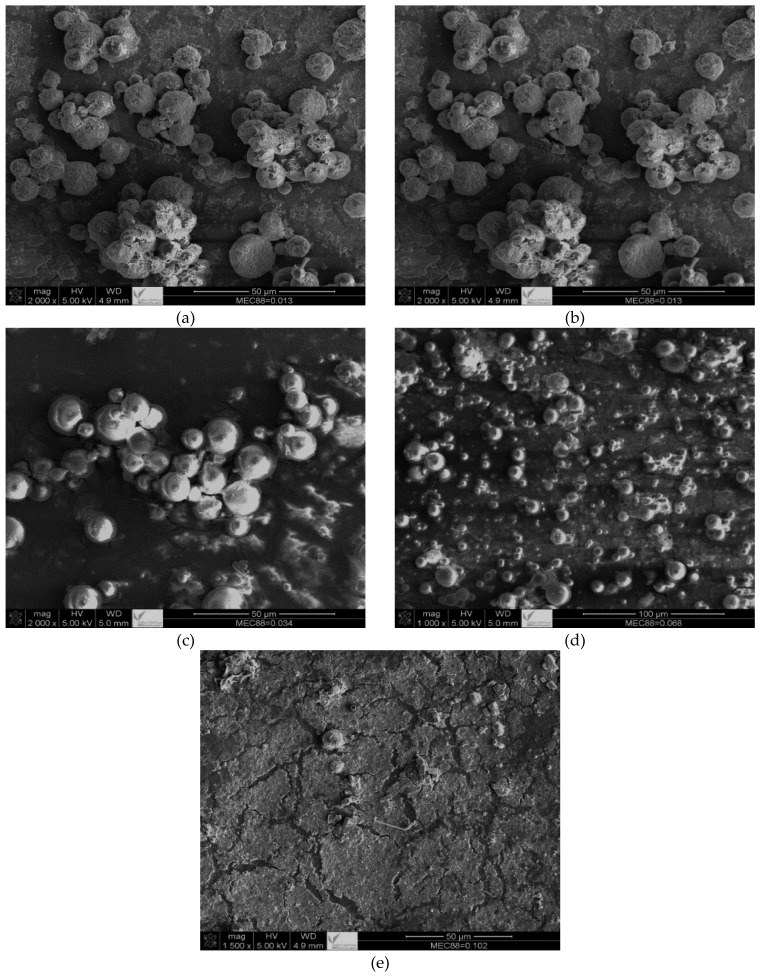
SEM micrographs of suspension polymerization of St and nBA in the presence of CNT-D and different concentrations of MEC-88: (**a**) SP-CNT-D-MEC-88-5; (**b**) SP-CNT-D-MEC88-4; (**c**) SP-CNT-D-MEC88-3; (**d**) SP-CNT-D-MEC88-2; and (**e**) SP-CNT-D-MEC88-1. The magnifications are 500× (scale bar = 50 µm) and 100× (scale bar = 100 µm).

The SEM micrographs presented in Figure 4 provide insights into why MEC-88 is not desirable for conversion into toner materials. Although the polymerized particles were in the micron range, the SEM micrographs show that they were not uniform, which is in agreement with the DLS results. Nonuniform particles also resulted in poor flowability, uneven charging, and reduced image quality, which are critical properties for toners. CCAs with positively charged surfaces and without carbonyl groups acting like positively charged metal oxides have been shown to solve issues regarding particle size and conversion [3,42,43]. SEM photographs of raw materials and polymerized toner can be found in the Supplementary Materials, Figures S1–S4.

### 3.3. Suspension Polymerization of Styrene and nBA in the Presence of CNT-D and MEP-51 in Different Concentrations

After obtaining unsatisfactory production of the materials for the toner using MEC-88, another attempt was made with MEP-51 (CCA type, principally positively charged, and no carbonyl group) to determine whether it could solve the retarding/inhibiting limitations of MEC-88. MEP-51 was chosen because of its role in improving toner properties, such as better transfer efficiency, reduced toner scatter, and higher print quality, by promoting the aggregation of toner particles and reducing their surface charge [44]. However, the main concern was determining the effect of MEP-51 on the conversion rate during toner production. As mentioned earlier, MEP-51 is soluble in neither styrene nor water, so it was first dissolved in acetone/ethanol in different amounts before being mixed with water (the water acting as a continuous phase containing PVA, SDS, and dispersed CNT-D).

When acetone was used, as mentioned in the experimental section, conversion of more than 45% was not achievable, and the greatest conversion was reached when the least amount of acetone was used. Previous studies indicate that CNT dispersion in acetone is not as good as its dispersion in water, so this result was expected because acetone is a polar aprotic solvent, implying that it has a low dielectric constant and cannot form hydrogen bonds. In contrast, water is a polar protic solvent, meaning that it can readily form hydrogen bonds. CNTs have hydrophobic surfaces because of sp^2^ hybridized carbon atoms [45], so they tend to aggregate in aqueous solutions. The difference in solubility of MEP-51 in water and acetone therefore impacts the reaction’s outcome, and acetone was thus found to be an unsuitable solvent for the conversion of monomers or the dispersion of CNT-D.

However, using ethanol in different amounts instead of acetone produced a promising conversion of 90% when a small amount (5 mL) of ethanol was used, so it was chosen as the solvent for dissolving MEP-51 before polymerization. Although ethanol and acetone are both polar solvents, they exhibit varying dielectric constants and hydrogen-bonding capabilities [46], so different solubility properties are to be expected. Ethanol dissolves MEP-51 better because it improves solubility and hydrophobic material dispersion through its ability to disrupt intermolecular forces and solvate the surfaces of the particles. The observed lower conversion rate of MEP-51 in acetone than in ethanol thus stems from acetone’s inability to adequately dissolve the monomer.

After selecting the solvent, the effect of MEP-51 concentrations on conversion and particle size was investigated. MEP-51 is a common CCA used in toner formulations to control the electrical charge of toner particles, which is necessary for proper printing. MEP-51 CCA is a quaternary ammonium compound that contains a long hydrocarbon chain, and its chemical structure allows it to adsorb onto the surface of toner particles, creating a negative charge. This negative charge helps to stabilize the toner particles and prevent them from clumping together. MEP-51 CCA can also help increase the toner’s sensitivity to magnetic fields, which is necessary for proper printing.

**Figure 5:**
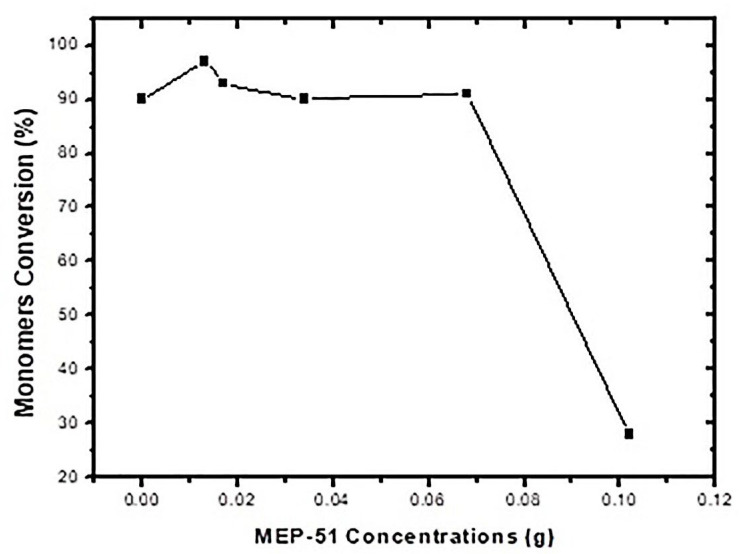
Monomer conversion versus MEP-51 concentration.

**Figure 6:**
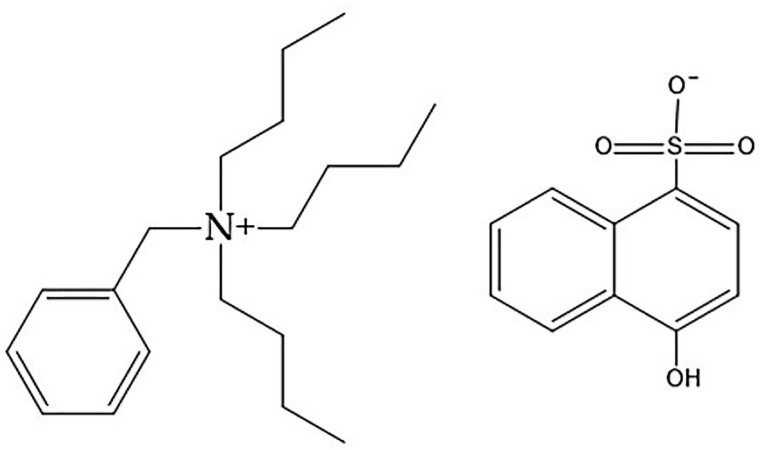
Chemical character of MEP-51.

As shown in Table 9 and Figure 5, the conversions were 90% or greater for all concentrations below 0.28 wt% (for the highest concentration, 0.42 wt%, the conversion was 28 wt%, which needs to be further investigated). This is in contrast to the findings for MEC-88. Simply put, greater MEP-51 concentrations resulted in higher conversion rates, with the two directly proportional, while the MEC-88 concentrations were inversely proportional to the conversion rates. This difference stems from the differences in their respective chemical characteristics: MEC-88 has carbonyl groups that have a retarding/inhibiting effect when suspension polymerization occurs, while MEP-51 lacks a carbonyl functional group, as shown in Figure 6.

**Figure 7:**
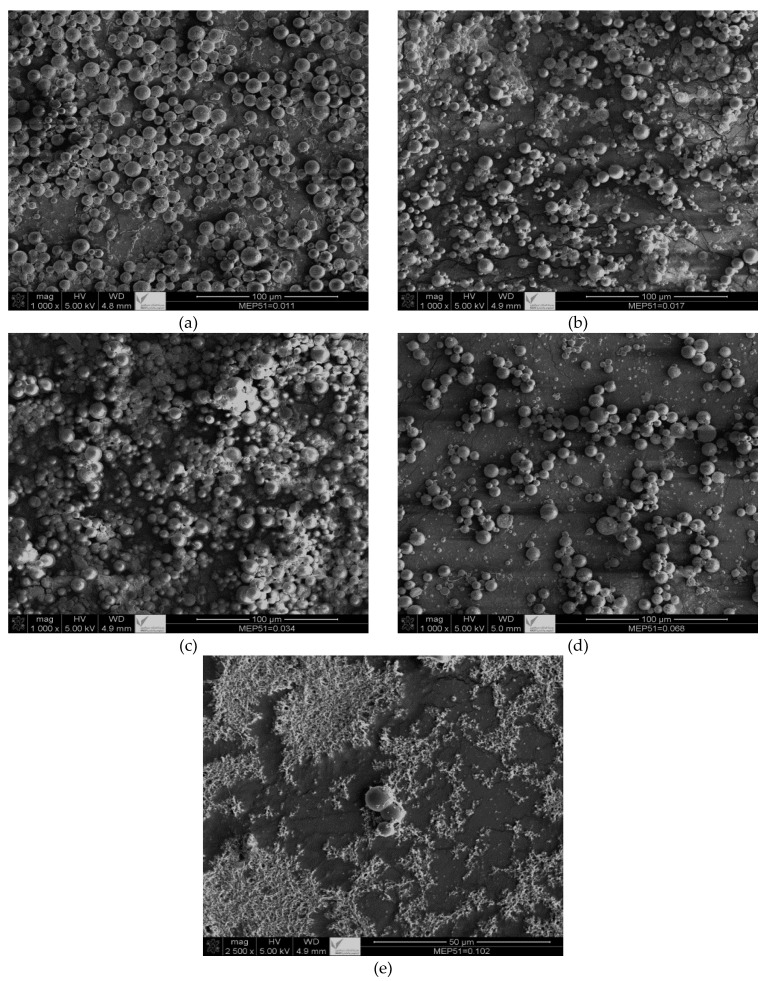
SEM micrographs for suspension polymerization of St and nBA in the presence of CNT-D and different concentrations of MEP-51: (**a**) SP-CNT-D-MEP-51-5; (**b**) SP-CNT-D- MEP-51-4; (**c**) SP-CNT-D- MEP-51-3; (**d**) SP-CNT-D- MEP-51-2; and (**e**) SP-CNT-D- MEP-51-1. The magnifications are 500× (scale bar = 50 µm) and 100× (scale bar = 100 µm).

According to Kiatkamjornwong et al. [26], the conversion of styrene and nBA (the same monomers as used in the present work) was not more than 80% in all cases in which carbon black (CB) was used as a pigment; the conversion decreased with greater CB concentrations. In contrast, in the present study, when CNTs were used, the conversion was found to be over 90%—more than 10% greater than with CB. This shows the advantages of using CNTs as a pigment instead of CB, which has never been reported before. Other advantages of using CNTs include the particle size of the toner materials: when it is small (of nm scale), the particles are undesirable because they can be harmful or unhealthy. The current study sought to develop new materials for a less harmful and more environmentally friendly toner, so the polymerized particle sizes for the toner materials were measurable in microns (see Figure 7).

There are four primary points shown in Figure 7: first, the toner particles remained within the micron range at all concentrations; second, the particle sizes obtained via SEM are consistent with the DLS measurements discussed in Table 9; third, the particle uniformity decreased with greater MEP-51 concentrations; and fourth, CNT particles were attached to the polymerized particles. Using CNTs as pigment in toner applications suggests that they behave similarly to CB. The advantages of CNTs over CB include high conversion and particle sizes in the micron range, which avoids the potential harm caused by particles in the nanometer range. In toner formulations, MEP-51 provides excellent control over the toner particles’ charge, ensuring that they are appropriately charged for efficient printing. As a result, the toner has better flowability, which is essential for even distribution during printing. In addition, MEP-51 improves toner adhesion to paper, producing sharp, high-quality prints.

## 4. Conclusions

The suspension polymerization of styrene and nBA in the presence of four CNTs (CNT-A nonmodified, CNT-B nonmodified with short chain, CNT-C modified with NH_2_, and CNT-D modified with boron) at different concentrations (varying between 0.065 wt% and 0.1 wt%) was investigated with the aim of selecting the best CNT for use as a pigment to produce particles for toner applications. Among those investigated, the only successful CNT was that modified with boron which achieved the best conversion and encapsulated particles for all concentrations. The remaining types were not effective enough to obtain high conversion or large particle sizes, even with very low CNT concentrations.

Various concentrations of the CNTs modified with boron (CNT-D) were investigated with the aim of selecting an optimum concentration for toner materials. Of the various CNT concentrations, 0.25 wt% showed good results concerning conversion, particle size, and encapsulation. CNT-D with a concentration of 0.25 wt% was used in the suspension polymerization of styrene and nBA in the presence of a CCA. Two types of CCA were tested: one dissolved in styrene (MEC-88), the other in acetone or ethanol (MEP-51). With MEC-88, it was not possible to reach a desirable conversion for all concentrations, and this is attributed to the retarding/inhibiting effects of mainly carbonyl groups. In contrast to the MEC-88 results, it was possible to achieve a high conversion rate (above 90%) with all concentrations of MEP-51 (except for the highest 0.1 wt%) when ethanol was used as the solvent instead of acetone, which did not show good conversion. Furthermore, SEM micrographs demonstrated that particle sizes were in the micron range, which was in agreement with DLS measurements, and that CNT attachment to the polymerized particles was good. The images demonstrated the uniformity of the particles achieved with the lowest concentration of MEP-51; as the concentration increases, particle uniformity decreases. In addition, the CNTs were seen to be attached to the cores of the particles.

## Figures and Tables

**Table 1:** Properties of graded CNTs used in suspension polymerization.

Sample Code	CNT Grade	Averages Diameter (nm)	Average Length (µm)	Carbon Purity (%)	Metal Oxide (%)	Amorphous Carbon	Surface Area (M^2^/g)
CNT-A	KNT-_L_ M31 (unmodified long MWCNT)	9.5	1.5	90	10	*	250–300
CNT-B	KNT-s M31 (unmodified short MWCNT)	3–10	0.5–2	>95	-	*	
CNT-C	KNT-MNH_13_ (MWCN modified with NH_2_)	13–18	1–12	99	-	*	N/A
CNT-D	KNT-MBO (MWCN modified with Boron)	20–30	N/A	N/A	-	*	N/A

* Pyrolytically deposited Carbon on the surface of the NC7000.

**Table 2:** Properties of two types of CCAs.

No.	Chemical Code	Charge µC/g
1	MEC-88	−20~−35
2	MEP-51	+30~+45

**Table 3:** Recipes for suspension polymerization of St and nBA in the presence of CNT-D and different concentrations of CCA (MEC-88).

Sample Code	Monomers		DVB (g)	BP (g)	PVA (g)	SDS (g)	CNT-D (g)	T (°C)	Time (h)	MEC-88		MEC-88:CNT-D	H_2_O (g)
	St (g)	nBA (g)								(g)	wt.%		
SP-CNT-D-MEC-88-1	18.18	4.49	1.828	0.25	0.25	0.046	0.034	75	7	0.102	0.42	3:1	150
SP- CNT-D-MEC-88-2	18.18	4.49	1.828	0.25	0.25	0.046	0.034	75	7	0.068	0.28	2:1	150
SP- CNT-D-MEC-88-3	18.18	4.49	1.828	0.25	0.25	0.046	0.034	75	7	0.034	0.14	1:1	150
SP- CNT-D-MEC-88-4	18.18	4.49	1.828	0.25	0.25	0.046	0.034	75	7	0.017	0.07	1:2	150
SP- CNT-D-MEC-88-5	18.18	4.49	1.828	0.25	0.25	0.046	0.034	75	7	0.013	0.05	1:3	150

**Table 4:** Recipes for suspension polymerization of St and nBA in the presence of CNT-D and CCA (MEP-51), with different amounts of acetone and ethanol used for dissolving MEP-51.

Sample Code	Monomers		DVB (g)	BP (g)	PVA (g)	SDS (g)	CNT-D (g)	Acetone (mL)	T (°C)	Time (h)	MEP-51		MEP-51:CNT-D	H_2_O (g)
	St (g)	nBA (g)									(g)	wt.%		
SP-CNT-D-MEP-51-Acetone-1	18.18	4.49	1.828	0.25	0.25	0.046	0.034	100	75	7	0.034	0.14	1:1	150
SP-CNT-D-MEP-51-Acetone 2	18.18	4.49	1.828	0.25	0.25	0.046	0.034	50	75	7	0.034	0.14	1:1	150
SP-CNT-D-MEP-51-Acetone 3	18.18	4.49	1.828	0.25	0.25	0.046	0.034	25	75	7	0.034	0.14	1:1	150
SP-CNT-D-MEP-51-Acetone-4	18.18	4.49	1.828	0.25	0.25	0.046	0.034	10	75	7	0.034	0.14	1:1	150
SP-CNT-D-MEP-51-Acetone-5	18.18	4.49	1.828	0.25	0.25	0.046	0.034	5	75	7	0.034	0.14	1:1	150
SP-CNT-D-MEP-51-Ethanol-1	18.18	4.49	1.828	0.25	0.25	0.046	0.034	100	75	7	0.034	0.14	1:1	150
SP-CNT-D-MEP-51-Ethanol-2	18.18	4.49	1.828	0.25	0.25	0.046	0.034	50	75	7	0.034	0.14	1:1	150
SP-CNT-D-MEP-51-Ethanol-3	18.18	4.49	1.828	0.25	0.25	0.046	0.034	25	75	7	0.034	0.14	1:1	150
SP-CNT-D-MEP-51-Ethanol-4	18.18	4.49	1.828	0.25	0.25	0.046	0.034	10	75	7	0.034	0.14	1:1	150
SP-CNT-D-MEP-51-Ethanol-5	18.18	4.49	1.828	0.25	0.25	0.046	0.034	5	75	7	0.034	0.14	1:1	150

**Table 5:** Recipes for suspension polymerization of St and nBA in the presence of CNT-D and different concentrations of CCA (MEP-51).

Sample Code	Monomers		DVB (g)	BP (g)	PVA (g)	SDS (g)	CNT-D (g)	Ethanol (mL)	T (°C)	Time (h)	MEP-51		MEP-51:CNT-D	H_2_O (g)
	St (g)	nBA (g)									(g)	wt.%		
SP-CNT-D-MEP-51-1	18.18	4.49	1.828	0.25	0.25	0.046	100	5	75	7	0.102	0.42	3:1	150
SP-CNT-D-MEP-51-2	18.18	4.49	1.828	0.25	0.25	0.046	50	5	75	7	0.068	0.28	2:1	150
SP-CNT-D-MEP-51-3	18.18	4.49	1.828	0.25	0.25	0.046	25	5	75	7	0.034	0.14	1:1	150
SP-CNT-D-MEP-51-4	18.18	4.49	1.828	0.25	0.25	0.046	10	5	75	7	0.017	0.07	1:2	150
SP-CNT-D-MEP-51- 5	18.18	4.49	1.828	0.25	0.25	0.046	5	5	75	7	0.013	0.05	1:3	150

**Table 6:** The effects of CNT types (CNT concentrations are constant at 0.5%) on suspension polymerization of St and nBA.

Samples	DLS Measurements		Conversions
	Z-Average	PDI	
CNT-A	625	0.6	9%
CNT-B	172	0.11	47%
CNT-C	195	0.159	58%
CNT-D	5715	0.35	96%

**Table 7:** The effects of CNT-A, CNT-B, CNT-C, and CNT-D concentrations on suspension polymerization of St and nBA.

Samples	CNT Concentrations (%)	DLS Measurements		Conversions
		Z-Average	PDI	
SP-CNT-A	0.065	228	0.3	76%
SP-CNT-A	0.125	1792	1.0	73%
SP-CNT-A	0.250	6621	0.4	70%
SP-CNT-A	0.500	625	0.6	9%
SP-CNT-A	0.750	1929	1.00	13%
SP-CNT-A	0.100	5749	0.84	10%
SP-CNT-B	0.065	1955	1.00	78%
SP-CNT-B	0.125	2557	1.00	72%
SP-CNT-B	0.250	1779	1.00	59%
SP-CNT-B	0.500	172	0.11	47%
SP-CNT-B	0.750	279	0.265	59%
SP-CNT-B	0.100	372	0.303	33%
SP-CNT-C	0.065	5216	0.176	75%
SP-CNT-C	0.125	782	0.617	73%
SP-CNT-C	0.250	151	0.255	75%
SP-CNT-C	0.500	195	0.159	58%
SP-CNT-C	0.750	1608	0.152	64%
SP-CNT-C	1.00	268	0.351	59%
SP-CNT-D	0.065	4259	1.00	96%
SP-CNT-D	0.125	4031	1.00	94%
SP-CNT-D	0.250	6621	0.4	90%
SP-CNT-D	0.500	5715	0.35	96%
SP-CNT-D	0.750	1929	1.00	93%
SP-CNT-D	1.00	5890	0.367	90%

**Table 8:** Suspension polymerization of St and nBA in the presence of CNT-D and five different concentrations of MEC-88.

Sample Code	CCA Con. (g)	CCA wt.%	CNT-D Con. (g)	CNT-D wt.%	Conversion (%)	DLS Measurement	
						Z-Average	PDI
SP-CNT-D-MEC-88-1	0.102	0.42	0.034	0.14	12	4032	1.00
SP-CNT-D-MEC-88-2	0.068	0.28	0.034	0.14	58	5882	0.16
SP-CNT-D-MEC-88-3	0.034	0.14	0.034	0.14	68	5077	0.50
SP-CNT-D-MEC-88-4	0.017	0.07	0.034	0.14	70	4576	1.00
SP-CNT-D-MEC-88-5	0.011	0.05	0.034	0.14	75	6910	0.57

**Table 9:** Suspension polymerization of styrene and butyl acrylate in the presence of CNT-D and five different concentrations of MEP-51.

Sample Code	CCA Con. (g)	CCA wt.%	CNT-D Con. (g)	CNT-D wt.%	Conversion (%)	DLS Measurement	
						Z-Average	PDI
SP-CNT-D-MEP-51-1	0.102	0.42	0.034	0.14	28	2106	1.00
SP-CNT-D-MEP-51-2	0.068	0.28	0.034	0.14	91	6793	0.226
SP-CNT-D-MEP-51-3	0.034	0.14	0.034	0.14	90	5251	0.146
SP-CNT-D-MEP-51-4	0.017	0.07	0.034	0.14	93	5784	0.217
SP-CNT-D-MEP-51-5	0.011	0.05	0.034	0.14	97	5882	0.161

## Data Availability

There are no linked research datasets for this submission. Data will be made available on request.

## Supplementary Materials

The following supporting information can be downloaded at: https://www.mdpi.com/article/10.3390/ma16113941/s1, Figure S1: Dispersion of CNT-A in water (left) and in chloroform (right), one week after sonication.; Figure S2: SEM micrographs of CNT-A dispersed in (a) Chloroform and (b) water.; Figure S3: SEM micrographs of SP-CNT-B at two different scales: (a) 2 µm and (b) 500 nm.; Figure S4. SEM micrographs of CNT-D attached to polymerized particles with two different concentrations (0.75% and 0.5%) and two different scales (10 and 5 µm).

## References

[1] Diamond A.S. (2001). Handbook of Imaging Materials.

[2] Kawamoto H., Nakayama N. (2016). Overview on recent progress in electrophotography. J. Imaging Sci. Technol..

[3] Ni W., Wu S., Ren Q. (2013). Silanized TiO_2_ nanoparticles and their application in toner as charge control agents: Preparation and characterization. Chem. Eng. J..

[4] Liu Z., Zhang Y., Pu T., Yuan X., Meng H., Yang H. (2017). Method for Preparing Suspension Polymerization Toner of Core-Shell Structure. U.S. Patent.

[5] Kmiecik-Lawrynowicz G.E., Patel R.D. (1995). Toner Emulsion Aggregation Process. U.S. Patent.

[6] AuClair C.J., Lu C.H. (1981). Emulsion Polymerization Process for Dry Positive Toner Compositions Employs charge control agent as wetting agent. U.S. Patent.

[7] Ober C.K., Lok K.P. (1986). Dispersion Polymerization Process for Toner Compositions. U.S. Patent.

[8] Bauer W., Lauth H. (1931). Patent (Ger.) Rohm and Hass, Darmstadt, Germany.

[9] Santos J.C., Lopes C., Reis M.M., Giudici R., Sayer C., Machado R.A.F., Araujo P.H.H. (2008). Comparison of techniques for the determination of conversion during suspension polymerization reactions. Braz. J. Chem. Eng..

[10] Schein L. (1999). Recent advances in our understanding of toner charging. J. Electrostat..

[11] Eren B., Solmaz Y. (2020). Preparation and properties of negatively charged styrene acrylic latex particles cross-linked with divinylbenzene. J. Therm. Anal. Calorim..

[12] Kopp S.-P., Düsenberg B., Eshun P.M., Schmidt J., Bück A., Roth S., Schmidt M. (2023). Enabling triboelectric charging as a powder charging method for electrophotographic powder application in additive manufacturing by triboelectric charge control of polymer particles. Addit. Manuf..

[13] Christie R. (2001). Colour Chemistry.

[14] Anderson J. (1996). The effect of additives on the tribocharging of electrophotographic toners. J. Electrostat..

[15] Park B., Hong S., Sim H., Choi H., Yoon Y. (2012). Effect of charge control agent on electrophoretic characteristics of polymer encapsulated titania nanoparticle. Mater. Chem. Phys..

[16] Baur R., Macholdt H.-T. (1993). Charge control agents for triboelectric (friction) charging. J. Electrostat..

[17] Fink J.K. (2011). Handbook of Engineering and Specialty Thermoplastics: Water Soluble Polymers.

[18] Fink J.K. (2017). A Concise Introduction to Additives for Thermoplastic Polymers.

[19] Visakh P., Thomas S. (2011). Handbook of Engineering and Specialty Thermoplastics, Polyethers and Polyesters.

[20] Michel E., Baur R., Macholdt H.-T. (2001). Charge stabilizers: Properties and applications. J. Electrostat..

[21] Düsenberg B., Kopp S.-P., Tischer F., Schrüfer S., Roth S., Schmidt J., Schmidt M., Schubert D.W., Peukert W., Bück A. (2022). Enhancing photoelectric powder deposition of polymers by charge control substances. Polymers.

[22] Lee L.-H. (1994). Dual mechanism for metal-polymer contact electrification. J. Electrostat..

[23] Higashima Y., Castle G., Inculet I., Brown J. (1993). The effect of an externally added charge control agent on contact charging between polymers. J. Electrostat..

[24] Nie M., Xia H., Wu J. (2013). Preparation and characterization of poly (styrene-co-butyl acrylate)-encapsulated single-walled carbon nanotubes under ultrasonic irradiation. Iran. Polym. J..

[25] Thakur A.K., Kurtyka K., Majumder M., Yang X., Ta H.Q., Bachmatiuk A., Liu L., Trzebicka B., Rummeli M.H. (2022). Recent advances in boron-and nitrogen-doped carbon-based materials and their various applications. Adv. Mater. Interfaces.

[26] Kiatkamjornwong S., Pomsanam P. (2003). Synthesis and characterization of styrenic-based polymerized toner and its composite for electrophotographic printing. J. Appl. Polym. Sci..

[27] Li C., Cheng W., Yan Z., Ge S., Shao Q., Naik N., Pan D., Guo Z. (2021). Soap-free styrene-acrylic/carbon nanotubes composite latex by in situ emulsion polymerization: Preparation, properties and characterizations. Surf. Interfaces.

[28] Park S.-H., Bandaru P.R. (2010). Improved mechanical properties of carbon nanotube/polymer composites through the use of carboxyl-epoxide functional group linkages. Polymers.

[29] Kim J.A., Seong D.G., Kang T.J., Youn J.R. (2006). Effects of surface modification on rheological and mechanical properties of CNT/epoxy composites. Carbon.

[30] Odian G. (2004). Principles of Polymerization.

[31] Abousalman-Rezvani Z., Eskandari P., Roghani-Mamaqani H., Salami-Kalajahi M. (2020). Functionalization of carbon nanotubes by combination of controlled radical polymerization and “grafting to” method. Adv. Colloid Interface Sci..

[32] Cho M.J., Choi D.H., Sullivan P.A., Akelaitis A.J., Dalton L.R. (2008). Recent progress in second-order nonlinear optical polymers and dendrimers. Prog. Polym. Sci..

[33] Hardy C.G., Zhang J., Yan Y., Ren L., Tang C. (2014). Metallopolymers with transition metals in the side-chain by living and controlled polymerization techniques. Prog. Polym. Sci..

[34] Hedayati H.R., Khorasani M., Ahmadi M., Ballard N. (2022). Preparation of well-defined Poly (Vinyl alcohol) by hydrolysis of Poly (Vinyl acetate) synthesized by RAFT suspension polymerization. Polymers.

[35] Chen Y., Wu Y., Li J., Peng X., Wang S., Jin H. (2022). Improving mechanical, electrical and thermal properties of fluororubber by constructing interconnected carbon nanotube networks with chemical bonds and f–h polar interactions. Polymers.

[36] Kedzior S.A., Dubé M.A., Cranston E.D. (2017). Cellulose nanocrystals and methyl cellulose as costabilizers for nanocomposite latexes with double morphology. ACS Sustain. Chem. Eng..

[37] Ataeefard M., Mohammadi Y., Saeb M.R. (2019). Intelligently synthesized in situ suspension carbon black/styrene/butylacrylate composites: Using artificial neural networks towards printing inks with well-controlled properties. Polym. Sci. Ser. A.

[38] Bhanvase B., Sonawane S. (2014). Ultrasound assisted in situ emulsion polymerization for polymer nanocomposite: A review. Chem. Eng. Process. Chem. Eng. Process..

[39] Zhang Y., Ye M., Han A., Ding C., Yang J., Zhang K. (2018). Preparation and characterization of encapsulated CoAl2O4 pigment and charge control agent for ceramic toner via suspension polymerization. Ceram. Int..

[40] Sai T., Ran S., Guo Z., Song P., Fang Z. (2022). Recent advances in fire-retardant carbon-based polymeric nanocomposites through fighting free radicals. SusMat.

[41] Maiti J., Basfar A.A. (2017). Encapsulation of carbon black by surfactant free emulsion polymerization process. Macromol. Res..

[42] Lin Y.-S., Hsu C.-F., Chen J.-Y., Cheng Y.-M., Lee P.-Y. (2016). Wear behavior of mechanically alloyed Ti-based bulk metallic glass composites containing carbon nanotubes. Metals.

[43] Otani S., Matsumoto Y., Takeuchi M. NIP & Digital Fabrication Conference. Proceedings of the Charging Mechanism of Polymers with CCA (II), Society for Imaging Science and Technology.

[44] Mizuguchi J., Sato Y., Uta K., Sato K. (2007). Benzyltributylammonium 4-hydroxynaphthalene-1-sulfonate. Acta Crystallogr. Sect. E Struct. Rep. Online.

[45] Sobolkina A., Mechtcherine V., Bellmann C., Khavrus V., Oswald S., Hampel S., Leonhardt A. (2014). Surface properties of CNTs and their interaction with silica. J. Colloid Interface Sci..

[46] Yoosefian M., Mola A. (2015). Solvent effects on binding energy, stability order and hydrogen bonding of guanine–cytosine base pair. J. Mol. Liq..

