# Supplementary material for: Encapsulation of Carbon Nanotubes by Styrene and Butyl Acrylate Particles via Suspension Polymerization for Polymerized Toner Applications
Source: Materials (Basel). 2023 May 24;16(11):3941. doi: 10.3390/ma16113941 (PMC10253727; doi:10.3390/ma16113941)
Supplement: Supplementary file 1 [file materials-16-03941-s001.zip › materials-2343039-supplementary.pdf]

materials

Supplementary Materials

Encapsulation of Carbon Nanotubes by Styrene and Butyl Acrylate Particles via Suspension Polymerization for Polymerized Toner Applications

Eid M. Alosime [1,*], Omar A. Adam [2] and Ahmed A. Basfar [3,4]

[1] King Abdulaziz City for Science and Technology, P.O. Box 6086, Riyadh 11442, Saudi Arabia
[2] Leibniz Institute of Polymer Research Dresden e.V., Hohe Straße 6, 01069 Dresden, Germany; omar.abakar.adam@gmail.com
[3] Mechanical Engineering Department, College of Engineering, King Saud University, P.O. Box 800, Riyadh 11421, Saudi Arabia; abasfar@ksu.edu.sa
[4] Nuclear Engineering Program, College of Engineering, King Saud University, P.O. Box 145111, Riyadh 11421, Saudi Arabia
* Correspondence: alosimi@kacst.edu.sa

A dispersion of multi-walled non-modified CNTs (CNT-A) in organic solvent with suitable Hansen parameters was investigated to compare the results obtained with water as the dispersed medium; however, from the appearances, there were no detectable differences in the dispersion state or the sedimentation of CNTs in water or in chloroform, even though the samples were kept for a week, as shown in Figure S1.

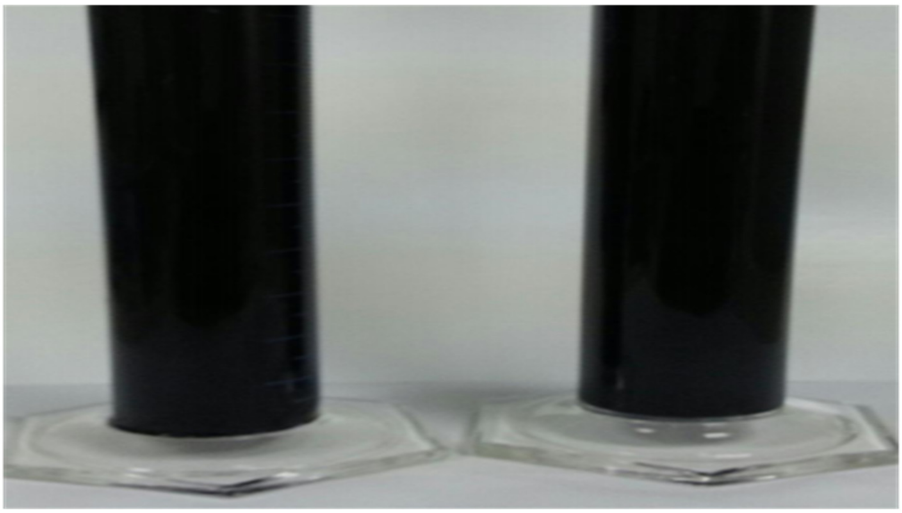

Figure S1. Dispersion of CNT-A in water (left) and in chloroform (right), one week after sonication.

In contrast to the above SEM micrographs in Figure S1, the dispersion state of CNT-A in water was better compared to that in chloroform, as presented in Figure S2. This finding supported using water as a suitable medium to achieve a good dispersion of CNTs. Furthermore, since the polymerization of monomers usually occurs in water media, there is a greater preference for using water instead of chloroform.

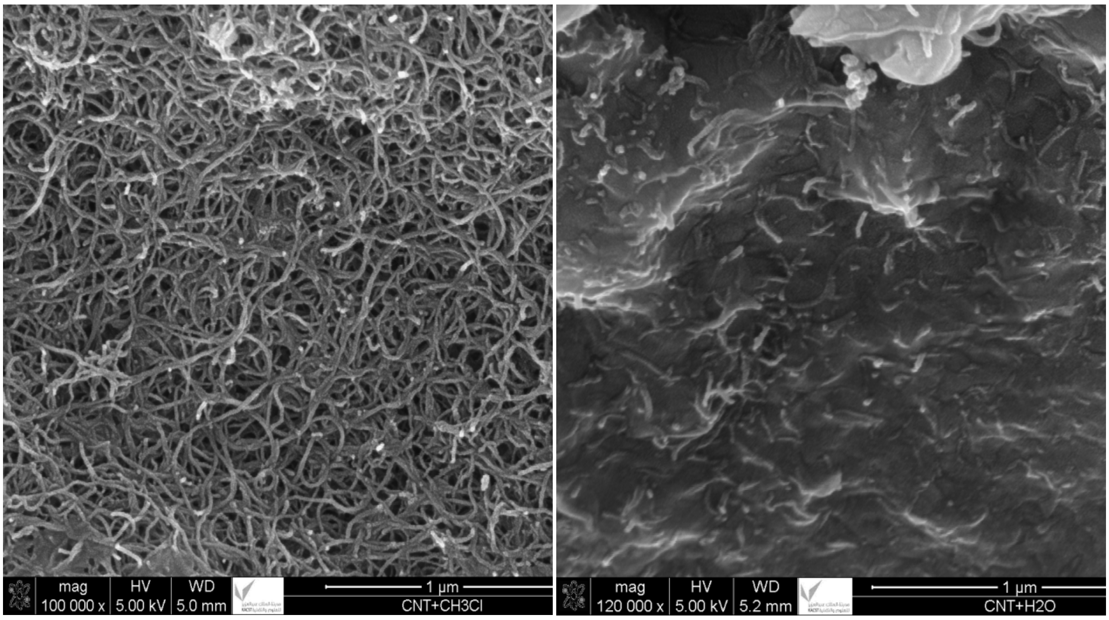

(a) (b)

Figure S2. SEM micrographs of CNT-A dispersed in (**a**) Chloroform and (**b**) water.

It is noticeable that the carbonyl group also affects the size of the polymerized particles and their shape, as shown in the SEM micrographs of CNT-B in Figure S3.

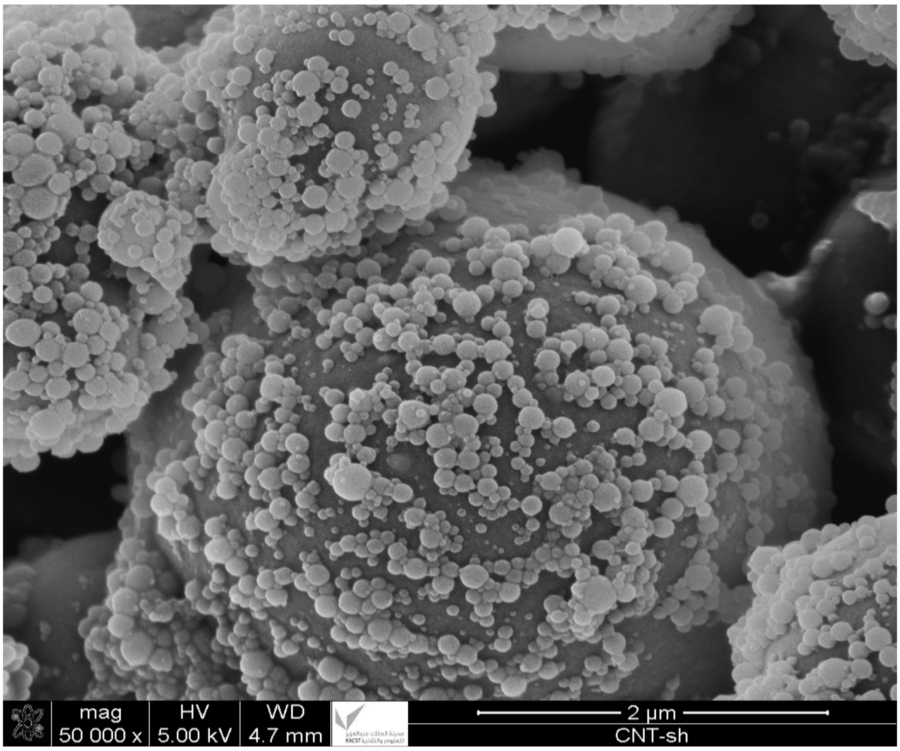

(a)

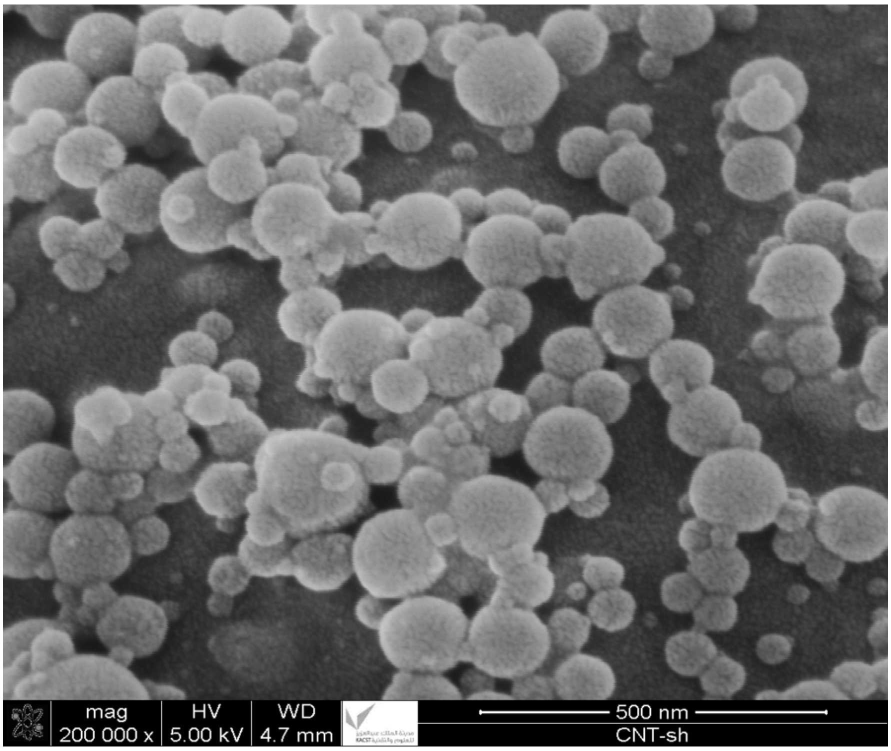

(b)

Figure S3. SEM micrographs of SP-CNT-B at two different scales: (**a**) 2μm and (**b**) 500 nm.

Regarding CNT-D (modified with Boron), this type of CNT demonstrated high conversion (90% and above). However, the SEM micrographs shown in Figure S4 for CNT-D 0.75 and 0.5wt% in polymerized particles make it clear that the attachment of CNT particles onto polymerized particles occurred. This finding proved that CNTs can perform the same behaviors as carbon black (CB). To our knowledge, CB is usually used in toner materials, in contrast to CNTs, which we need to be aware of when they are used in toner materials.

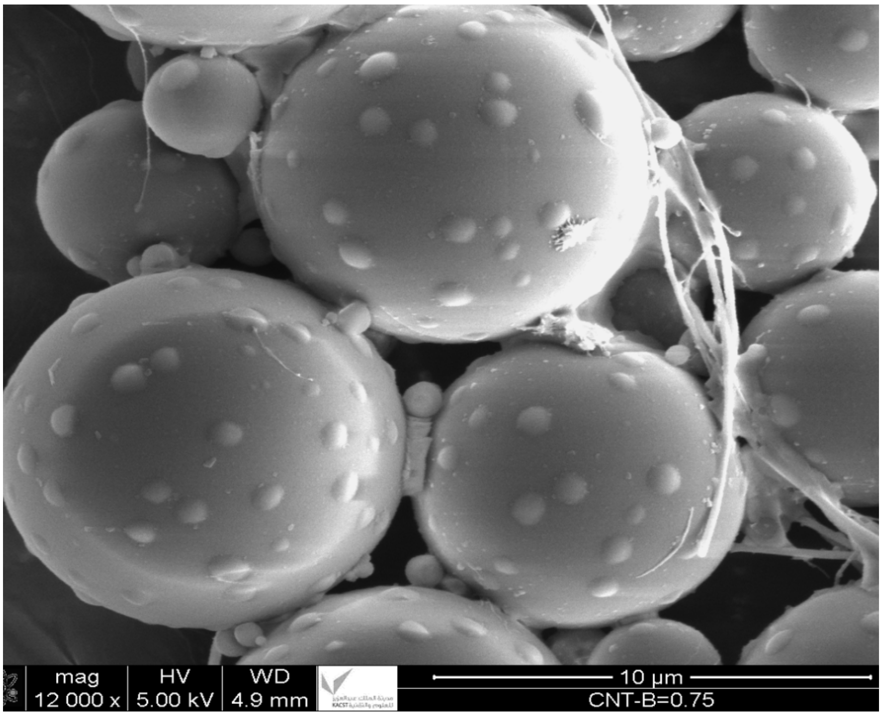

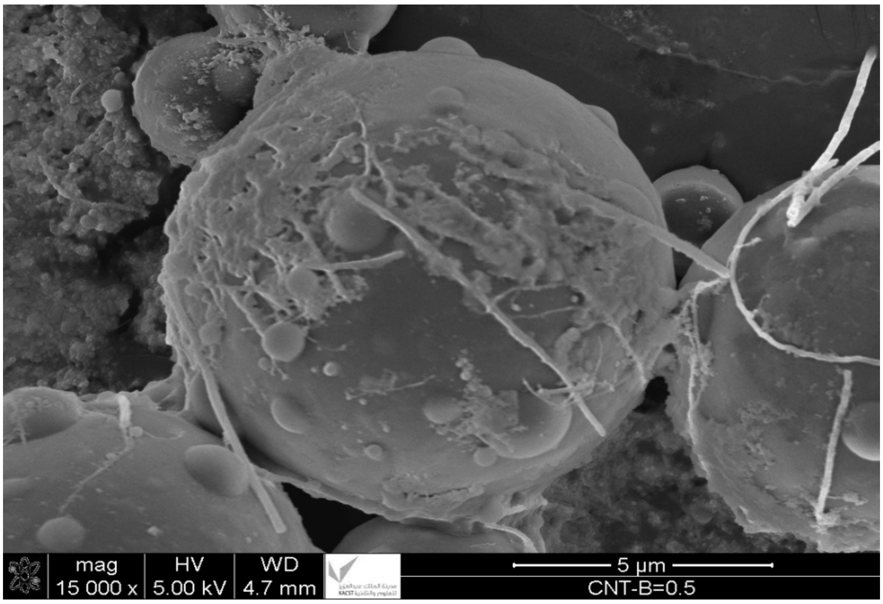

Figure S4. SEM micrographs of CNT-D attached to polymerized particles with two different concentrations (0.75% and 0.5%) and two different scales (10 and 5 µm).

Disclaimer/Publisher's Note: The statements, opinions and data contained in all publications are solely those of the individual author(s) and contributor(s) and not of MDPI and/or the editor(s). MDPI and/or the editor(s) disclaim responsibility for any injury to people or property resulting from any ideas, methods, instructions or products referred to in the content.
